# Oxaliplatin desensitization for ovarian **cancer** in pregnancy: A case report

**DOI:** 10.1016/j.gore.2024.101354

**Published:** 2024-02-28

**Authors:** Kaitlin Nicholson, Lily Jia, Margaret Rowe, Katharine Esselen, Naima Joseph, Chloe A. Zera, Timothy Lax, Meghan Shea

**Affiliations:** a Department of Obstetrics and Gynecology, Beth Israel Deaconess Medical Center, Boston, MA, United States; b Harvard Medical School, Boston, MA, United States; c Department of Pharmacy, Beth Israel Deaconess Medical Center, Boston, MA, United States; d Department of Medicine, Penn Medicine, Philadelphia, PA, United States; e Division of Maternal Fetal Medicine, Department of Obstetrics and Gynecology, Boston Medical Center/Boston University, Boston, MA, United States; f Department of Medicine, University of Massachusetts Memorial Health, Worcester, MA, United States; g Department of Medicine, Beth Israel Deaconess Medical Center, Boston, MA, United States

**Keywords:** Ovarian cancer, Hypersensitivity reaction, Desensitization, Platinum chemotherapy

## Abstract

• Incidence of cancer in pregnancy is rising and successful treatment of these patients requires expert multidisciplinary care.
• Platinum hypersensitivity reactions in ovarian cancer are commonly treated with desensitization protocols.
• To our knowledge, chemotherapy desensitization in pregnant patients has not been previously reported.
• Oxaliplatin desensitization during pregnancy may be safe and feasible.

## 1 Introduction

Cancer diagnoses affect approximately 1/1000 pregnancies (Smith et al., 2003). There has been an increase in incidence over recent decades in countries where delayed childbearing is common and where noninvasive prenatal testing is more accessible, the latter of which increases the detection of asymptomatic cancers (Amant et al., 2019). It is estimated that ovarian cancer occurs in 1 in 10,000 pregnancies, the majority of which are stage I, low-grade, and germ cell or noninvasive epithelial histology (Salani et al., 2014).

Management of ovarian cancer in pregnancy is dependent upon the distribution of disease at the time of diagnosis, the timing of gestation, and the desire to continue the pregnancy (Amant et al., 2019). In cases where a pregnancy is continued, often a combination of uterine-sparing cytoreductive surgery (preferably in the second trimester) and systemic therapy is recommended for advanced epithelial ovarian cancers (Amant et al., 2019). The most commonly used systemic regimens are platinum-based and have been shown to be safe with minimal increased maternal toxicity, pregnancy-related complications, or adverse neonatal outcomes (Amant et al., 2019). Systemic therapy is typically given in the second and third trimester and withheld within three weeks of delivery to avoid risks of maternal and neonatal myelosuppression (Amant et al., 2019, Salani et al., 2014).

Chemotherapy induced hypersensitivity reactions (HSR) are a well-known complication of cancer treatment. While there are four types of drug HSRs, type I reactions are the most common HSR seen with ovarian cancer. Type I HSR involve drug-specific Immunoglobulin E (IgE) antibody and antigen cross-linking with mast cells and basal cells, causing the immediate release of histamine and tryptase. The release of these inflammatory mediators results in the classic signs and symptoms, including, flushing, pruritis, urticaria/angioedema, rash, tachycardia, sense of impending doom, changes in blood pressure, dyspnea, desaturation, throat tightness, congestion, coughing, gastrointestinal upset, or neurologic disturbances (Picard et al., 2014). This clinical picture can also occur through IgE independent mechanisms (Castells et al., 2008).

Platinum-based chemotherapy such as carboplatin, cisplatin, and oxaliplatin can cause hypersensitivity typically through type I HSRs; the incidence increases with the number of platinum infusions (Picard et al., 2014). HSRs to cisplatin, carboplatin, and oxaliplatin occur in 5–20 %, 1–44 %, and 10–19 % of patients, respectively (Rassy et al., 2023, Makrilia et al., 2010). While desensitization protocols have been described for these chemotherapeutic agents in nonpregnant patients, the literature on desensitization during pregnancy is sparse and mostly related to antibiotics and other medications (Castells et al., 2008, Rassy et al., 2023, Gastaminza et al., 2011, Banjar et al., 2023, Garcia et al., 2021, Kuhlen et al., 2015).

Special considerations must be taken when attempting drug desensitization during pregnancy, including fetal monitoring and drug dosing based on pharmacodynamic and pharmacokinetic changes seen in pregnancy, given the additional risk of a severe hypersensitivity reaction and possible fetal compromise, including neurologic damage or death (Banjar et al., 2023, Carra et al., 2021). Hypersensitivity reactions during desensitization have been reported in 24–56 % of patients, 6–20 % being moderate-to-severe in nature (Castells et al., 2008). Preparation for desensitization in pregnancy should include a multidisciplinary team of medical oncologists, gynecologic oncologists, high-risk obstetricians, pharmacists, allergists, anesthetists, and neonatologists (Banjar et al., 2023). Here, we present our experience with a patient who had advanced epithelial (mucinous) ovarian cancer during pregnancy and underwent oxaliplatin desensitization in her third trimester.

## 2 Case report

The patient is a 30-year-old G2P1001 who presented with abdominal pain and was diagnosed with an 8.7 cm x 5.8 cm complex right adnexal mass and an elevated CA-125 to 76 U/mL during pregnancy. An Magnetic resonance imaging (MRI) of the pelvis revealed a large complex cystic lesion of the right adnexa with a mural nodule, suspicious for a cystic ovarian neoplasm.

At 19 weeks of gestational age, she underwent an exploratory laparotomy, right salpingo-oophorectomy, infracolic omentectomy, appendectomy, and peritoneal biopsies with a gynecologic oncologist. Intraoperatively, sanguineous ascites, evidence of pre-operative cyst rupture, and two palpable tumor implants less than 1 cm over the lower anterior abdominal wall were found. The appendix appeared grossly normal, as did the upper gastrointestinal tract. At the end of the procedure, all visible disease was resected.

**Fig. 1:**
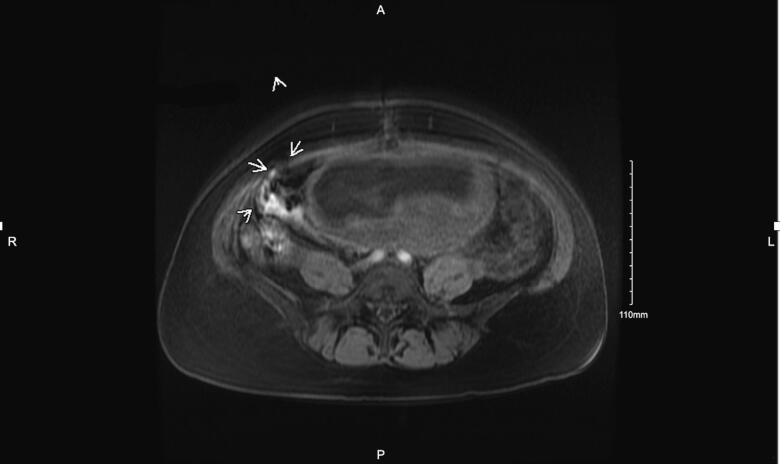
Magnetic resonance imaging (MRI) of abdomen after initial fertility-sparing staging surgery showing a soft tissue implant in the right lower quadrant, consistent with residual disease.

Pathology revealed a grade 2 mucinous adenocarcinoma of the right fallopian tube/ovary, also involving a right anterior abdominal wall implant (CK7 + strong/diffuse; CK20 + focally, CDX2 + focally; negative for PAX8, ER, SATB2). Both infiltrating and expansile types of invasion were present. The appendix was without evidence of dysplasia or malignancy. No lymph nodes were submitted. Thus, she was diagnosed with FIGO Stage IIIB, grade 2 mucinous adenocarcinoma of the ovary. A postoperative MRI noted soft tissue nodule implants in the right lower quadrant concerning for residual peritoneal disease (Fig. 1). No lymphadenopathy was noted. Her CEA was 0.5 ng/mL and CA 19–9 was 11 U/mL. Endoscopy and colonoscopy revealed no evidence of malignancy.

The patient subsequently underwent four two-week cycles of 5-fluorouracil and oxaliplatin (FOLFOX) with curative intent. During her fourth cycle of oxaliplatin infusion, she began to feel itchy and her palms and chest were noted to be red. Oxaliplatin was stopped and she was given intravenous fluids, intravenous famotidine 20 mg, and intravenous diphenhydramine 25 mg. She appeared to recover and subsequently received 5-fluorouracil push with infusion. After ambulation, she again appeared red felt itchy, nauseous, and short of breath and received another 25 mg IV diphenhydramine. She was then monitored for 30 min with resolution of symptoms and went home connected to 5-FU pump without issues. Tryptase and IL6 levels were not elevated at the time of the reaction. She was seen by an allergy specialist and oxaliplatin skin testing was negative.

Nonetheless, given the severity of the patient’s reaction and concern for a more severe anaphylactic reaction that could lead to early delivery and adverse neonatal outcomes, her allergist recommended a 16-step, four-bag Oxaliplatin desensitization protocol. In addition, her maternal fetal medicine (MFM) physician recommended a course of steroids for fetal lung maturity at 33 weeks of gestation in case of spontaneous or indicated preterm delivery. To facilitate the safe administration and both maternal and fetal monitoring during the desensitization process, she was admitted to the inpatient labor and delivery service. She was instructed to take oral cetirizine 10 mg and oral montelukast 10 mg the night before admission. Once admitted, she was pre-medicated with oral cetirizine 10 mg, oral montelukast 10 mg, intravenous methylprednisolone 80 mg, intravenous ondansetron 16 mg, intravenous diphenhydramine 50 mg, and oral famotidine 40 mg prior to the first desensitization bag.

Throughout the infusion and for two hours afterwards, she was placed on continuous telemetry and fetal monitoring, with blood pressure assessments every 15 min. The 4-bag desensitization protocol involved the first bag at 1/1000th dilution, followed by the second and third bag at 1/100th dilution and 1/10th dilution, respectively. The fourth and last bag was delivered at the standard dilution of 85 mg/m^2^. The rate was titrated every 15 min as tolerated to a maximum rate of 80 ml per hour (Table 1).

**Table 1:** Oxaliplatin desensitization protocol.

**Standard volume per bag**	250 mL		
**Final rate of infusion**	80 mL/h		
**Calculated target concentration**	0.62 mg/mL		
**Standard time of infusion**	120 min		


	**Volume per bag (mL)**	**Dilution**	**Total dose per bag (mg)**
**Solution 1**	9.3	1/1000	0.155
**Solution 2**	9.3	1/100	1.55
**Solution 3**	18.8	1/10	15.5
**Solution 4**	250	Standard	155

**4-bag desensitization**			
**Steps**	**Solution**	**Time per step (min)**	**Dose administered per step (mg)**
1–4	1	15	0.155
5–8	2	15	1.55
9–12	3	15	15.5
13–16	4	15	155

**3-bag desensitization**			
**Steps**	**Solution**	**Time per step (min)**	**Dose administered per step (mg)**
1–4	2	15	1.55
5–8	3	15	15.5

After the completion of six antepartum cycles of FOLFOX with oxaliplatin desensitization for cycles 5 and 6 (33 and 35 weeks gestation), she had an MRI abdomen/pelvis that showed resolution of the right lower quadrant implant.

She then had a planned cesarean section at 37 weeks and 5 days of gestation, followed by immediate completion interval cytoreductive surgery with planned abdominal cesarean hysterectomy, left salpingo-oophorectomy, omentectomy, peritoneal biopsies, and resection of nodules from anterior abdominal wall. Intraoperative findings were notable for scarce millimetric white nodules and adhesions; there were two firm scarred nodules in the right abdominal sidewall and in the right anterior abdominal wall. All were resected and sent to pathology. There was otherwise no visible disease and total estimated blood loss was 1000 mL. There was no residual malignancy in any of the specimens on final pathology. Her postoperative course was uncomplicated and she was discharged home with her infant on post-operative day five. She was recommended against breastfeeding. The infant was in the median percentiles for both weight and head circumference and did not have any postnatal complications.

**Fig. 2:**
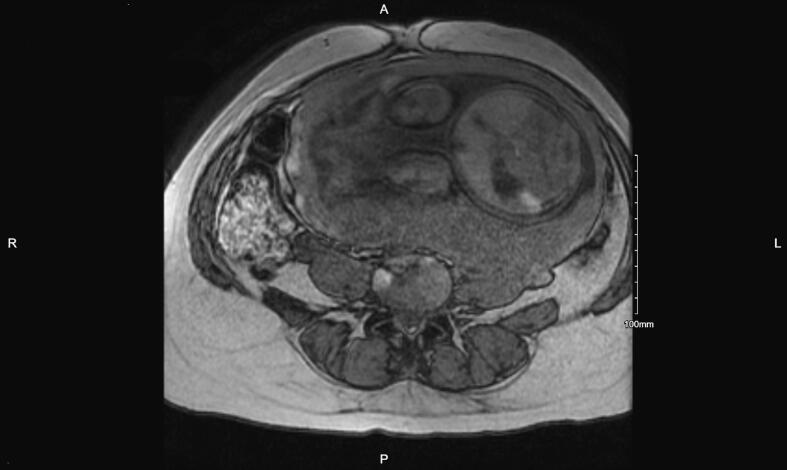
Antepartum MRI abdomen/pelvis showing resolution of right lower quadrant implant after FOLFOX.

Approximately five weeks post-partum after concurrent cesarean delivery and interval cytoreductive surgery, the patient resumed the remaining six cycles of FOLFOX with oxaliplatin desensitization for a total of twelve cycles. Since she tolerated the 4-bag desensitization protocol without complications, she was transitioned to a 3-bag protocol postpartum for cycles 8–12. Her post-treatment imaging showed no evidence of disease and tumor markers remained normal. At twelve months follow-up, the patient continues on surveillance without evidence of disease (Fig. 2). Genetic testing did not show any cancer-predisposing mutations in any of the 84 genes tested.

## 3 Discussion

This case describes a patient diagnosed with advanced epithelial ovarian cancer, specifically a mucinous adenocarcinoma, during pregnancy who received FOLFOX with oxaliplatin desensitization after the development of an HSR. The diagnosis of cancer during pregnancy is rare and additional treatment considerations are required to achieve an optimal obstetrical, neonatal, and oncological outcome (Amant et al., 2019). Maternal fetal outcomes of pregnancies complicated by cancer include, non-viable pregnancy, growth restriction, preterm delivery and associated consequences, and neonatal death (Salani et al., 2014). However, the administration of taxanes, platinum agents, anthracyclines, etoposide, and bleomycin (amongst others) has proven to be feasible and safe in the second and third trimesters (Amant et al., 2019).

HSRs are a relatively common occurrence in patients receiving platinum-based therapies, particularly after the sixth treatment (Picard et al., 2014). Skin testing can be used to assess risk of IgE mediated HSR (Pagani et al., 2022). In a cohort of patients with gynecologic cancer undergoing retreatment with carboplatin, skin testing had an 81 % to 98.5 % negative predictive value and 86 % positive predictive value for developing HSR (Pagani et al., 2022). However, skin testing for oxaliplatin has a wider range of reported negative predictive values, ranging from 26 % to 100 % (Gorgulu Akin et al., 2022). Given this variation, it is not surprising that this patient’s skin testing was negative for an oxaliplatin reaction, yet clinically consistent with HSR based on her reaction.

There are published desensitization protocols for platinum chemotherapies; these generally involve premedication with antihistamines and steroids followed by slow infusion of a dilute solution of the target drug (Castells et al., 2008). The rate of infusion and drug concentration increase at regular intervals until reaching the standard rate and concentration. One common desensitization protocol starts with a 100-fold dilution of the final concentration, transition to a 10-fold dilution solution, and a final undiluted solution (Castells et al., 2008). In cases when the patient is at increased risk of more profound HSR (i.e., in this case of desensitization in pregnancy), occasionally a 4th dilution (1000-fold dilution) is added in. The concentration of the final solution is calculated by subtracting the cumulative dose administer in prior steps from the target dose. The infusion rate of each solution is increased every fifteen minutes for one hour before transitioning to the next solution.

To our knowledge there are no other reports of chemotherapy desensitization in pregnancy but there are some reports of antibiotic desensitization protocols. In one report, a pregnant patient with multiple drug allergies underwent a 12-step drug desensitization protocol to Ceftaroline, taking about five hours to complete (Kuhlen et al., 2015). The desensitization procedure was conducted in an intensive care unit with oral and parenteral steroids and as-needed antihistamines and epinephrine.

In this report, we describe a case of curative intent treatment with FOLFOX and oxaliplatin desensitization in patient diagnosed with advanced mucinous epithelial ovarian cancer in pregnancy. By utilizing a multidisciplinary approach with medical oncology, gynecologic oncology, maternal-fetal medicine, pharmacy, anesthesia, and allergy, a safe and feasible desensitization protocol was carried out without complication, resulting in the delivery of a healthy infant at term and a favorable oncologic outcome.

## 4 Conclusion

Drug desensitization in pregnancy poses unique challenges, and anaphylactic reactions can result in placental hypoperfusion with negative consequences for the fetus (Garcia et al., 2021). It is important to carefully consider the risks and benefits, both maternal and fetal, regarding first-line and alternative therapies prior to proceeding with desensitization. In this report, oxaliplatin desensitization in pregnancy was safe and feasible. In light of the rising incidence of malignancy in pregnancy, protocols for drug desensitization in pregnant patients may become increasingly necessary and further study is needed to determine the optimal approach for this patient population.

## CRediT authorship contribution statement

**Kaitlin Nicholson:** Writing – review & editing, Writing – original draft, Project administration, Data curation, Conceptualization. **Lily Jia:** Writing – review & editing, Writing – original draft, Data curation, Conceptualization. **Margaret Rowe:** Writing – review & editing, Writing – original draft. **Katharine Esselen:** Writing – review & editing, Supervision, Conceptualization. **Naima Joseph:** Writing – review & editing. **Chloe A. Zera:** Writing – review & editing. **Timothy Lax:** Writing – review & editing. **Meghan Shea:** Writing – review & editing, Validation, Supervision, Conceptualization.

## Declaration of competing interest

The authors declare that they have no known competing financial interests or personal relationships that could have appeared to influence the work reported in this paper.
